# Episodic memory improvements due to noninvasive stimulation targeting the cortical–hippocampal network: A replication and extension experiment

**DOI:** 10.1002/brb3.1393

**Published:** 2019-09-30

**Authors:** Molly S. Hermiller, Erica Karp, Aneesha S. Nilakantan, Joel L. Voss

**Affiliations:** ^1^ Interdepartmental Neuroscience Program, Department of Medical Social Sciences Ken and Ruth Davee Department of Neurology Department of Psychiatry and Behavioral Sciences, Feinberg School of Medicine Northwestern University Chicago IL USA

**Keywords:** associative memory, episodic memory, noninvasive brain stimulation, rTMS

## Abstract

**Introduction:**

The distributed cortical network of the human hippocampus is important for episodic memory. In a previous experiment, noninvasive stimulation of the hippocampal‐cortical network applied for five consecutive days improved paired‐associate learning measured after the stimulation regimen via cued recall (Wang et al., *Science*, 2014, **345**, 1054). This finding has not yet been directly replicated. Furthermore, evidence for long‐lasting effects of stimulation on paired‐associate learning was obtained by analyzing relatively small subsamples (Wang & Voss, *Hippocampus*, 2015, **25**, 877) and requires further evaluation.

**Methods:**

Sixteen healthy young adults participated in this replication study using the same experimental design as the original study. Participants received 1 week of active stimulation and 1 week of sham stimulation, with memory assessments occurring at the beginning (pre) and end (post) of each week. Assessments included the paired‐associate task used in the original study, as well as a long‐term episodic memory retention task in order to test the hypothesis that increased paired‐associate learning could come at the cost of accelerated long‐term forgetting. Change in memory scores was evaluated within (pre vs. post) and across (active vs. sham) weeks.

**Results:**

Similar to Wang et al., paired‐associate learning was significantly improved after 1 week of active stimulation but not after 1 week of sham stimulation. We found no evidence that stimulation increased long‐term forgetting for either week.

**Conclusion:**

These findings confirm the beneficial effects of stimulation on episodic memory that were reported previously and indicate that stimulation‐related gains in new learning ability do not come at the price of accelerated long‐term forgetting.

## Graphical abstract

In a previous experiment, noninvasive stimulation of the cortical–hippocampal network applied for five consecutive days improved paired‐associate learning measured after the stimulation regimen via cued recall. Here, we replicated the experimental design in 16 healthy young adults and similarly found significant improvement in paired‐associate learning due to stimulation. Furthermore, we found no evidence that stimulation‐related gains in new learning ability come at the expense of accelerated long‐term forgetting.

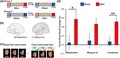

## 1 INTRODUCTION

The distributed network of the human hippocampus includes regions such as the medial prefrontal, medial parietal, and lateral parietal cortex and is thought to support episodic memory (Buckner, Andrews‐Hanna, & Schacter, 2008; Mesulam, 1990; Ranganath & Ritchey, 2012). Several experiments have tested contributions of the hippocampal–cortical network to memory using noninvasive brain stimulation (Hermiller, VanHaerents, Raij, & Voss, 2019; Kim et al., 2018; Nilakantan, Bridge, Gagnon, VanHaerents, & Voss, 2017; Nilakantan et al., 2019; Tambini, Nee, & D'Esposito, 2018; Wang et al., 2014). These experiments have used a network targeting approach, whereby transcranial magnetic stimulation (TMS) was applied to cortical regions defined based on their resting‐state fMRI connectivity with the hippocampus. The effects of stimulation on the targeted hippocampal–cortical network have been measured using memory testing and neuroimaging. There have been several demonstrations of improved recollection memory performance (Kim et al., 2018; Nilakantan et al., 2017, 2019; Tambini et al., 2018; Wang et al., 2014) as well as corresponding changes in neuroimaging‐based measures of network function (Hermiller et al., 2019; Kim et al., 2018; Nilakantan et al., 2019; Wang et al., 2014). These experiments have therefore been important for identifying the role of hippocampal–cortical networks in memory.

These previous experiments have used different episodic memory outcomes, including cued recall of face‐word associates (Wang et al., 2014), spatial reconstruction (Nilakantan et al., 2017; Tambini et al., 2018), and paired‐associate recognition (Kim et al., 2018; Nilakantan et al., 2019). Findings that stimulation improved performance for these various test formats, all of which measure hippocampal‐dependent recollection, provide important conceptual replications of the phenomenon of improved episodic memory due to stimulation targeting the hippocampal–cortical network. However, replication using the same memory outcome is important for a variety of reasons, including testing whether anyone finding is robust to the variety of factors that can differ across experiments and robust given sample size (Button et al., 2013). The primary goal of the current experiment was to replicate the seminal report in this area (Wang et al., 2014), using the same face‐word paired‐associate recall memory test from that experiment. Notably, this replication was performed using different MRI scanners, different TMS systems, and a different data collection team relative to Wang et al. (2014), thereby providing an evaluation of whether the effect of network‐targeted stimulation on face‐word recall task performance is robust to these methodological details.

One aspect of the Wang et al. (2014) findings that especially warrants further investigation is that stimulation produced long‐lasting memory improvement (Wang & Voss, 2015). The experiment involved a within‐subjects crossover design, whereby stimulation was applied during one week and sham the other, in counterbalanced order across subjects, and with pre‐/postmemory testing performed for each week. There was an approximately 2‐week washout period, and subgroup analysis suggested that the memory benefits due to stimulation extended across the washout period (Wang & Voss, 2015). That is, subjects receiving stimulation during their first week continued to show elevated recall performance when they returned and completed the preweek test for the sham week, relative to subjects receiving sham during their first week. Although this finding of long‐lasting stimulation effects is of potential theoretical and practical importance, the relevant analysis involved reduced power, as the total sample had to be split into halves based on stimulation/sham delivery order.

The current experiment also sought to address potential mechanisms whereby network‐targeted stimulation improves memory. The effects of network‐targeted stimulation on memory demonstrated in previous experiments (Kim et al., 2018; Nilakantan et al., 2017, 2019; Tambini et al., 2018; Wang & Voss, 2015) have been improvements in new learning ability. In these studies, the memory assessment involved learning a novel set of memoranda and then taking the memory test after a relatively brief delay (minutes to hours) on the same day. Thus, the effects of stimulation on memory reflected changes in the ability to acquire and successfully express memory for these novel memoranda. These previous experiments have not tested whether stimulation also affects the retention of memoranda learned prior to the delivery of stimulation. Indeed, there is potentially a tension between learning of new information and retention of old information via factors such as retroactive interference (Wixted, 2004), retrieval‐induced forgetting (Anderson, 2003), and/or increased neurogenesis (Davis & Zhong, 2017; Frankland, Kohler, & Josselyn, 2013; Richards & Frankland, 2017). It is therefore possible that enhanced learning via stimulation could come at the cost of accelerated forgetting. Proposed hippocampal neurogenesis‐based mechanisms of the learning–forgetting tradeoff are of particular interest, as TMS may promote hippocampal neurogenesis (Guo, Lou, Han, Deng, & Huang, 2017). Thus, if stimulation were to enhance new learning while also accelerating long‐term forgetting of material learned prior to stimulation, these mechanisms would be implicated. To evaluate this possibility, we tested the effects of stimulation on 5‐day retention of word‐pair associates learned at the beginning of each experimental week (before stimulation delivery) and tested at the end of each experimental week (after the 5‐day stimulation regimen).

## 2 METHODS

### 2.1 General overview

**Figure 1:**
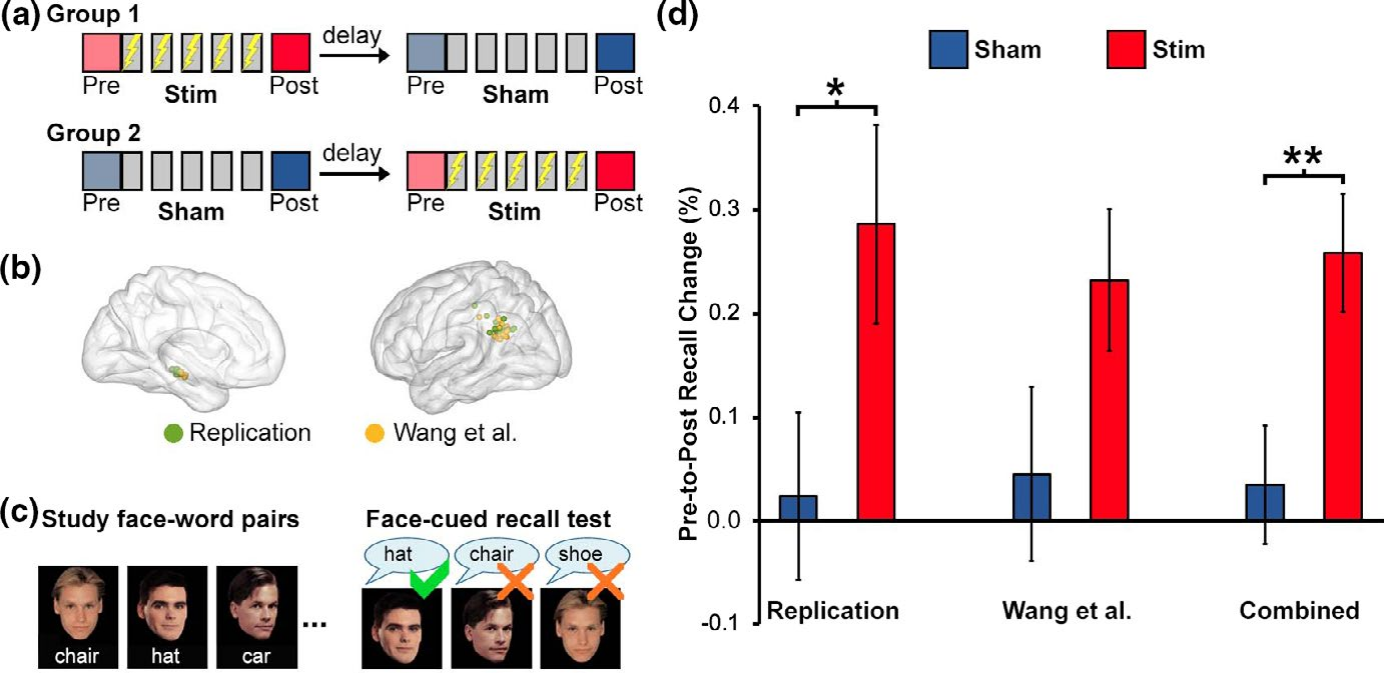
(a) Half of the subjects received stimulation daily for the first week and sham daily for the second week (Group 1), with the order reversed for the remaining subjects (Group 2). Assessments were given immediately before the first stimulation session (Pre) and ~24 hr after the final stimulation session (Post). (b) The hippocampal locations used as seeds for resting‐state fMRI connectivity analysis (left) and the parietal locations selected as stimulation targets (right) based on high resting‐state fMRI connectivity with the hippocampus were highly similar to locations in the Wang et al. (2014) experiment. (c) The face‐word paired‐associate recall task was administered as in Wang et al. (2014). (d) As in Wang et al. (2014), there was significant improvement in face‐cued word recall due to stimulation relative to sham in the current replication experiment. Furthermore, this improvement was evident when using data pooled across experiments. Statistical values are not indicated for the Wang et al. (2014) results, which are plotted here as a reference, in order to avoid redundant statistical reporting. *p < .05. **p < .005

This experiment used a within‐subjects counterbalanced design with two separate weeks of experimental procedures separated by a delay period (Figure 1a). Subjects received five consecutive daily sessions of stimulation targeting the hippocampal–cortical network during one week and five consecutive daily sessions of sham stimulation to the same location during the other week, with the order of conditions (stimulation/sham) counterbalanced across subjects. Each week included a baseline test (Pre‐Stim or Pre‐Sham) administered approximately 1 hr before the first TMS session and a follow‐up test (Post‐Stim or Post‐Sham) administered approximately 24 hr after the final/fifth TMS session. The face‐word paired‐associate recall task that measured new learning was administered on each of these four assessments using different stimuli per assessment. The verbal paired‐associate task that measured long‐term forgetting was administered for each week, with the study period occurring during baseline (Pre‐Stim or Pre‐Sham) and the retention test administered during the corresponding follow‐up assessment (Post‐Stim or Post‐Sham). As in our previous experiments, TMS was delivered to parietal locations of the hippocampal–cortical network selected according to baseline resting‐state fMRI connectivity with the hippocampus (Figure 1b). Subjects also completed memory tasks during fMRI scanning at each assessment as well as a battery of neuropsychological measures, and findings from these other measures were reported previously (Kim et al., 2018).

### 2.2 Methodological similarities and differences from Wang et al. (2014)

The experiment design followed that of Wang et al. (2014) and the same face‐word paired‐associate task was used. Subjects performed a lengthy battery of in‐scanner and out‐of‐scanner memory and cognition tasks as part of both experiments, such that subjects could not have easily guessed the specific tasks that we hypothesized would benefit from stimulation. The current study delivered the first stimulation session of each week about 1 hr after the baseline test (Pre‐Stim or Pre‐Sham). In Wang et al. (2014), the first stimulation session was 1 day after the Preassessment. The washout period between conditions for this current study was an average of 10.1 weeks (range = 4–30 weeks), whereas Wang et al. (2014) had a mean washout period of 2.5 weeks (range = 1–4 weeks). Although baseline resting‐state fMRI connectivity was used to identify stimulation locations as in Wang et al. (2014), the current study used a Siemens PRISMA scanner (see below) whereas Wang et al. (2014) used a Siemens TRIO scanner, with different resting‐state fMRI scan parameters and different analysis steps in the two experiments (see below). Stimulation was performed using a MagVenture X100 TMS system in the current experiment (see below), whereas a NexStim TMS system was used in Wang et al. (2014). The same TMS intensity calibration method and stimulation parameters were used in both experiments. Although both experiments used standard “figure‐8” coils, they differed in geometry and therefore in the properties of their induced fields. Finally, the Wang et al. (2014) data were collected primarily by a postdoctoral fellow (the first author of the report) who closely supervised efforts of a small number of research assistants, whereas the current data were collected by a large team of six full‐time research assistants and their staff managers as well as three graduate students. Although most experiments do not consider the composition of the individuals involved in data collection, this is a potentially important variable that differed substantially between our original report and the current replication attempt.

### 2.3 Participants

Data are reported for *N* = 16 adults (12 females, mean age 25.81 years, range 18–34 years). Data from different tasks for these same subjects are reported in Kim et al. (2018). These subject characteristics are approximately the same as for Wang et al. (2014) (*N* = 16, 9 females, mean age 24 years, range 20–32 years), and subjects in both experiments were recruited from the downtown Chicago area and paid for their participation.

### 2.4 Face‐word paired‐associate recall test

The face‐word paired‐associate recall task was the same as described in Wang et al. (2014). In brief, for each assessment, subjects sequentially viewed 20 faces printed on cards for approximately 3 s each. Each face was paired with a spoken and arbitrarily paired common noun. After a filled delay of approximately one minute, subjects were shown the same 20 faces in a different random order and were required to recall the word that had been paired with each face. Accuracy was computed as the number of faces for which the correct/matching word was produced (production of words that were studied but incorrectly paired to the current face were counted as incorrect). A different set of faces and words was used for each of the four assessments (Pre‐Stim, Pre‐Sham, Post‐Stim, and Post‐Sham). Two subjects received a computerized version of the same task, which was identical except that the faces were presented on a computer screen and the words played as audio files instead of spoken by the experimenter. Details concerning the faces, words, and other aspects of administration can be found in Wang et al. (2014).

Two subjects were removed from the current dataset for having outlier change values, which was defined as an increase or decrease in performance from one assessment to another of more than 2 *SD* from the group average change, which was equivalent to an increase or decrease of 100%. One of the subjects removed showed an outlier increase in performance due to stimulation and the other showed an outlier decrease due to stimulation, and so outlier removal was not biased toward the predicted improvement. For analyses that used data combined from the current experiment and from the Wang et al. (2014) experiment, the same outlier threshold was used for the Wang et al. (2014) data. This resulted in removal of one subject from the Wang et al. (2014) dataset, who showed outlier increase in performance due to stimulation. Thus, outlier removal was overall against the predicted improvement effect (two removed for outlier increases and one removed for outlier decrease).

### 2.5 Long‐term forgetting test

A verbal paired‐associate task was used to assess long‐term forgetting. Subjects studied 28 pairs of arbitrarily matched and unrelated common words using a retrieval practice format (Sumowski et al., 2010) in order to achieve above‐chance retention across the 5‐day study–test delay. The study phase was administered at baseline (Pre‐Stim or Pre‐Sham), and the corresponding retention test was administered on the corresponding follow‐up assessment (Post‐Stim or Post‐Sham, respectively).

The study phase comprised 112 trials, with each trial including either reading (28) or retrieval practice (84). Each of the 28 verbal paired associates (VPAs) was presented four times, twice in a reading format and twice in a retrieval practice format. The order of the two words within a VPA was consistent across trials (i.e., always BOOK‐STUDY, never STUDY‐BOOK). During a reading trial, subjects read the two words aloud. For retrieval practice, only the first word of the pair was presented and the subject had to verbally recall the second word. Feedback was immediately given, whereby the experimenter said either “correct” or “incorrect” and then read the correct response aloud. The first and third presentation of each VPA was a reading trial, with retrieval practice trials following after a randomized delay of 1–4 trials. The second reading trial occurred 7–85 trials after the first retrieval practice. Thirty seconds after the study phase, the final study test was administered. For this, the first word of each pair was presented, and subjects had to recall the second word, and no feedback was given. Accuracy was computed as the number of trials for which correct word pairings were recalled, with a possible range of 0–28. The delayed retention test followed the same format as the final study test. Forgetting was calculated as the difference in the number of paired associates recalled during the delayed retention test versus the corresponding final study test.

There were two versions of the verbal paired‐associate task that used different words, and the words did not overlap with those used in the face‐word paired‐associate task. One version was administered for the stimulation week and one for the sham week, with counterbalanced assignment of test version to stimulation condition. VPAs for each version are provided as Table A1. There were no subjects with outlier change values on this test, and so no exclusions were made.

### 2.6 MRI and TMS methods

Resting‐state fMRI and structural MRI were used to identify the target location for TMS delivery. Full details of the MRI scanning parameters, preprocessing, and target identification were reported in Kim et al. (2018). In brief, an individual location of the hippocampal body was identified for each subject and used as a seed for resting‐state fMRI connectivity analysis. For each subject, a stimulation target was identified based on maximal resting‐state fMRI connectivity values with the hippocampal seed constrained within a region of parietal cortex that is typically considered part of the hippocampal–cortical network. Despite differences in MRI scanners, MRI scan parameters, preprocessing, and analysis details between Wang et al. (2014) and the current experiment, the locations of hippocampus used as connectivity seeds and the locations of parietal cortex selected as stimulation targets were highly similar (see Section 3).

Transcranial magnetic stimulation was performed using one of two identical MagVenture X100 systems with stereotactic guidance using subject‐specific MRI. Full details of stimulation are described in Kim et al. (2018). In brief, stimulation involved 1,600 total pulses of 20‐Hz rTMS delivered in 40 2‐s trains (40 pulses at 20Hz per train), with an intertrain interval of 28 s. Stimulation intensity was calibrated to 100% of resting motor threshold, with intensity lowered slightly for comfort for 6/16 subjects. On average, stimulation was delivered at 95% resting motor threshold (range = 80%–100%, *SD* = 7.9). Sham was delivered using the same parameters but at 10% of resting motor threshold, delivered to the same target location as stimulation.

For subjects in the current experiment, the estimated TMS‐induced e‐fields were modeled using the forward‐modeling Boundary Element Method (Nummenmaa et al., 2013). Full localization data were not available for two subjects. The mean peak intensity for modeled subjects (14/16) was 86.3 V/m (*SD* = 16.9). E‐fields for the Wang et al. sample were estimated in real‐time using a spherical head model by the TMS navigation software (Nexstim Ltd.). The mean peak intensity for the Wang et al. subjects was 83.7 V/m (*SD* = 19.0). There was no significant difference in mean peaks during active stimulation between samples (*t*(28) = 0.85; *p* = .40).

### 2.7 Analysis

Percentage‐change scores were calculated to measure the change in memory performance for each week of stimulation relative to its baseline (i.e., Post‐Stim vs. Pre‐Stim; Post‐Sham vs. Pre‐Sham). Repeated‐measures analysis of variance (RM‐ANOVA) and paired two‐tailed *t* tests were used to compare mean performance levels for each condition as well as percentage‐change scores. Effect sizes are reported as partial eta squared ($\eta_{p}^{2}$) and Cohen's *d*.

## 3 RESULTS

The hippocampal targets and parietal stimulation locations were similar in the current replication sample as in the Wang et al. sample (Figure 1b). To quantify the consistency of parietal cortex stimulation targeting across samples, we computed Euclidean distances for each subject's stimulation location in the replication sample versus all other subjects' locations in the replication sample (i.e., 15 values per subject; 240 distance values total in the sample; mean = 13.6 mm, *SD* = 7.3 mm), as well as the mean distances for each subject's location in the replication sample versus all subjects’ locations in the Wang et al. sample (i.e., 15 values per subject; 240 values total in the sample; mean = 12.5 mm, *SD* = 8.2 mm). The distance values did not significantly compute among samples in the current dataset versus computed between the current dataset and the Wang et al. dataset (*t*(478) = 1.7, *p* = .10). Further, the numerical trend of lower distances for locations in the replication sample to locations in the Wang et al. (2014) sample suggests that replication sample stimulation locations were closer to those in Wang et al. than they were to other locations within the replication sample. Thus, despite methodological differences, stimulation targeting the hippocampal–cortical network was applied similarly in the replication sample as in Wang et al. (2014).

In the replication sample, face‐cued word recall performance increased significantly due to stimulation relative to prestimulation baseline (28.6% improvement; *t*(13) = 2.98, *p* = .01), whereas there was no significant increase for sham relative to presham baseline (2.3% improvement; *t*(13) = 0.29, *p* = .78). The increase due to stimulation was significantly greater than the increase due to sham (*t*(13) = 2.55, *p* = .02, Cohen's *d* = 0.68) (Figure 1). The same pattern of findings was obtained in the replication sample when calculated using raw values rather than percentage‐change scores (Table 1), which showed significant interaction between session (prestimulation/poststimulation) and stimulation condition (active/sham) in RM‐ANOVA [*F*(1,52) = 5.79; *p* < .03; $\eta_{p}^{2}$ = 0.31]. This overall pattern replicates the Wang et al. (2014) findings, which are shown for reference in Figure 1d, calculated using the current methods and without corresponding statistical tests in order to avoid duplicate reporting of Wang et al. findings.

**Table 1:** Face‐word paired‐associate recall performance

	Stim			Sham			Stim(Post–Pre) versus Sham (Post–Pre)
	Pre	Post	Post–Pre	Pre	Post	Post–Pre	
Replication	8.4 (0.7)	10.6 (1.0)	2.2 (0.7)*	10.6 (0.9)	10.7 (1.1)	0.1 (0.7)	2.1 (0.9)*
Combined	8.8 (0.5)	10.7 (0.6)	1.9 (0.4)**	10.6 (0.7)	10.6 (0.7)	0.0 (0.5)	1.9 (0.6)**

* *p* < .05.

** *p* < .005.

Combining data from the replication and Wang et al. (2014) samples, there was a 25.8% increase in performance due to stimulation (*t*(28) = 4.49, *p* < .001) and no significant increase due to sham (3.4% increase; *t*(28) = 0.60, *p* = .55). The increase for stimulation was significantly greater than that for sham (*t*(28) = 3.14, *p* = .004, Cohen's *d* = 0.58). The same pattern of findings was obtained in the combined data when calculated using raw values rather than percentage‐change scores (Table 1), which showed significant interaction between session (prestimulation/poststimulation) and stimulation condition (active/sham) in RM‐ANOVA [*F*(1,112) = 9.84; *p* < .004; $\eta_{p}^{2}$ = 0.26]. Overall, these findings indicate that the effects of stimulation (relative to sham) on face‐cued word recall performance were similar across studies, yielding “medium” effect sizes in both samples and when pooling data across samples.

As in the Wang et al. dataset, there was a significant difference in baseline performance for the stimulation and sham weeks such that prestimulation performance was lower than presham performance (Table 1). This was evident for the replication sample (*t*(13) = 2.64, *p* = .02) and for the combined data (*t*(28) = 3.37, *p* = .002). As was explored in Wang and Voss (2015), one potential reason for this difference in baseline performance is that there could have been an ordering/carry‐over effect, whereby the influence of stimulation on performance persisted and thereby affected the presham performance value. Wang and Voss (2015) addressed this by sorting subjects into those who received stimulation first (i.e., Group 1) versus those who received sham first, (i.e, Group 2), as this was a within‐subjects design with counterbalanced order. However, given that the sample was only *N* = 16, this yielded only *n* = 8 per group. Using the combined data, we were able to analyze the stimulation order effect with a larger sample size. We used raw scores for this analysis (rather than percentage‐change) as it is easier to appreciate the longitudinal effects across multiple consecutive sessions using raw scores.

**Figure 2:**
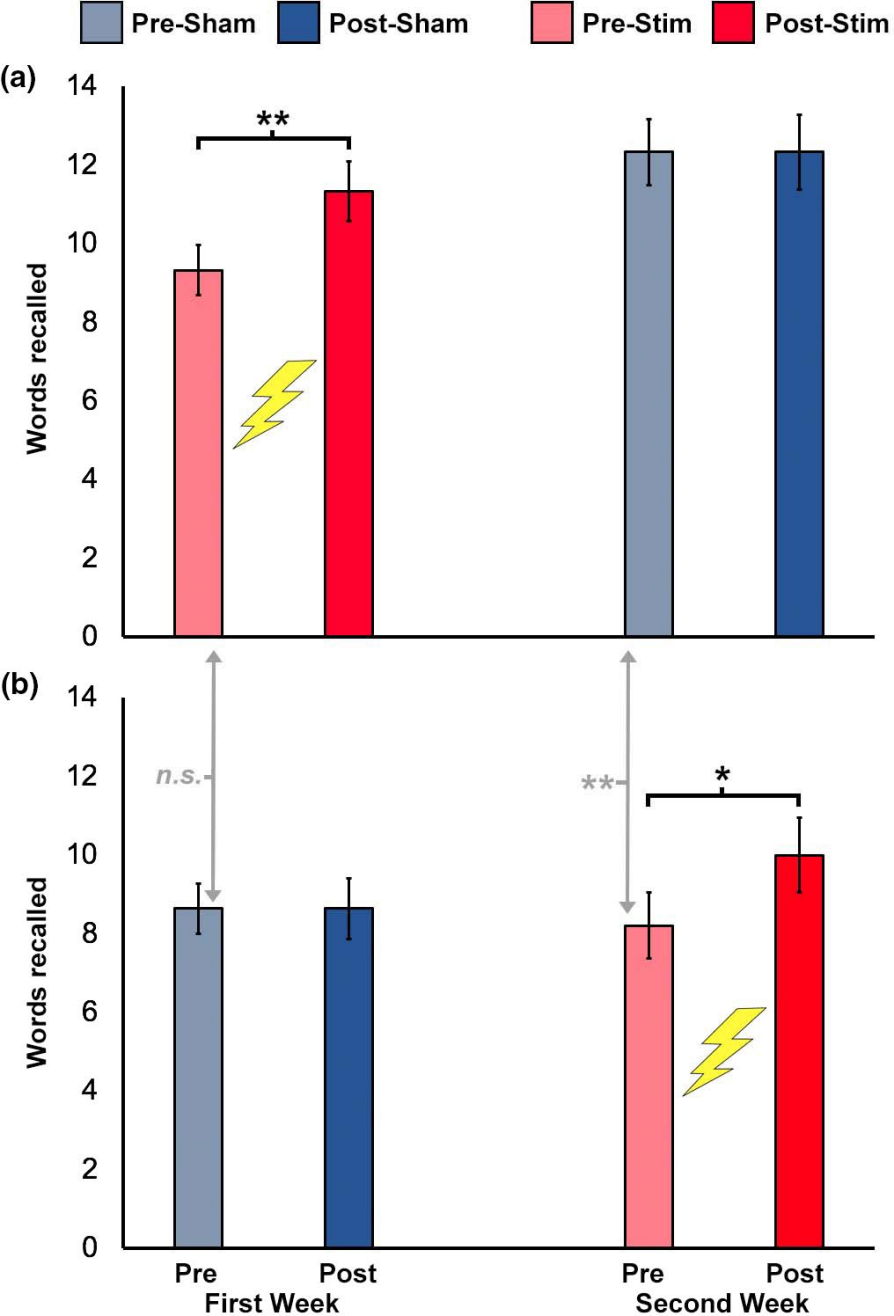
Face‐cued word recall performance is plotted separately for the two groups of subjects that differed based on whether they received stimulation during the first week (a) or sham during the first week (b). The timing of stimulation delivery is indicated by the lightning bolt symbol. Gray arrows extending between panels A and B indicate statistical tests between groups. *p < .05. **p < .01. n.s. nonsignificant

When ordering was taken into account, there was not a baseline difference in performance between groups for the first test administered (prestimulation Group 1 mean = 9.33 vs. presham Group 2 mean = 8.64; *t*(27) = 0.62, *p* = .54), indicating that the baseline performance difference described above was likely due to ordering effects. Group 1 showed significant improvement prestimulation versus poststimulation during the first week (*t*(14) = 3.62, *p* = .003), and then no subsequent change over the subsequent sham week (*t*(14) = 0.0, *p* = 1.0), with consistently elevated performance after the initial boost due to stimulation (Figure 2a). Group 2 showed effectively the same pattern of positive response to stimulation and null response to sham, with no significant change across the first week of sham (*t*(13) = 0.0, *p* = 1.0), and then a significant improvement due to stimulation (*t*(13) = 2.83, *p* = .01) (Figure 2b).

As was the case in Wang and Voss (2015), the persistence of the improvement due to stimulation was significant. This was tested by comparing the presham value for Group 1 to the prestim value for Group 2 (i.e., the performance level on the first day of the second week for subjects in Group 1 who received stimulation during the first week compared to the first day of the second week for subjects in Group 2 who received sham during the first week), as indicated by the dashed line in Figure 2a,b; *t*(27) = 3.49, *p* = .002). The increase in performance due to stim (prestim to poststim) was marginally greater for subjects receiving stim in the first week compared to subjects receiving sham in the first week (*t*(27) = 2.02, *p* = .05), indicating that stimulation was marginally more effective for improving memory scores without first having a week of sham. Thus, the ordering effect reported in Wang and Voss (2015) was identified in this larger, combined sample.

To test the assumption that enhanced learning of new information comes at the expense of accelerated forgetting of previously learned information (Davis & Zhong, 2017; Frankland et al., 2013; Richards & Frankland, 2017), the retention of word‐pair associates learned at the beginning of each week and tested at end of each week were compared between stimulation conditions. The rate of word‐pair associate long‐term forgetting across the week‐long stimulation regimen was not significantly influenced by stimulation. Performance levels were roughly matched by the end of the learning phase for Pre‐Stim (mean = 21.6, *SE* = 1.4) and Pre‐Sham (mean = 21.8, *SE* = 1.5) sessions, and subjects recalled approximately the same number of words at the Post‐Stim (mean = 7.0, *SE* = 1.3) and Post‐Sham (mean = 8.2, *SE* = 1.5) sessions. The number of words forgotten (Preperformance minus Postperformance) did not differ for the Stim and Sham conditions (*t*(15) = 0.54, *p* = .60). When forgetting was calculated as a percentage of the number of words‐pair associations successfully learned at baseline, there was no significant difference in forgetting for Stim (mean = 70.0%, *SE* = 4.4%) than for Sham (mean = 62.7%, *SE* = 5.4%) (*t*(15) = 1.50, *p* = .15). Thus, stimulation did not affect the retention of learned word‐pair associates across the week. The stimulation‐induced improvement in face‐word paired‐associate learning (Figure 2) was therefore not accompanied by enhanced long‐term forgetting of word‐pair associates.

## 4 DISCUSSION

The beneficial effects of stimulation targeting the hippocampal–cortical network for learning face‐word paired associates were nearly identical in this replication experiment as in Wang et al. (2014). Although the same general stimulation method was followed and the same face‐cued word recall test was administered, the replication experiment used a different TMS system, different MRI scanners and fMRI parameters, and was conducted by a different type of data collection team than the original report. Thus, the effects of stimulation on episodic memory replicated despite these methodological differences. Notably, the magnitude of the memory improvement was very similar in the replication experiment as in the original experiment and was robust when combining data from both experiments, with similar standardized effect sizes for both experiments and when computed using the combined data.

In the current experiment, we also tested the effect of stimulation on long‐term forgetting of verbal paired associates. Paired‐associate lists were studied prior to the five‐day stimulation regimen and tested afterward. We did not find reliable change in long‐term forgetting due to stimulation. Thus, stimulation did not seem to affect the potential tradeoff between new learning and long‐term forgetting that has been suggested by experiments investigating the effects of neurogenesis on episodic memory (Davis & Zhong, 2017; Richards & Frankland, 2017) or due to other factors (Anderson, 2003; Wixted, 2004). This suggests that the beneficial effects of stimulation on new learning occur via a different mechanism.

A notable aspect of the replication findings is that the effects of stimulation on face‐word paired‐associate learning persisted long after the stimulation period, consistent with the original experiment (Wang & Voss, 2015). Notably, the primary memory outcome was tested approximately 24 hr after the final stimulation session, and therefore, all effects of stimulation on learning reported here and in the original report were relatively persistent. The surprisingly long‐lasting effects were identified by analyzing the maintenance of improvement over the several‐week washout period that was inserted between the stimulation and sham weeks. That is, as in Wang and Voss (2015), we found an ordering effect, whereby subjects receiving stimulation during the first week continued to demonstrate elevated performance for their second week (of sham), relative to subjects receiving sham during the first week (Figure 2). Importantly, subjects receiving stimulation during the first week did not differ at baseline (i.e., the first testing session) from subjects receiving sham during the first week. This indicates that there were no general group differences in performance. Likewise, subjects receiving sham during first week did improve, only after receiving stimulation during the second week (Figure 2). The long‐lasting improvement across the between‐week period therefore reflects the effects of stimulation rather than group differences or other nonspecific factors, and the current experiment verified this finding with a larger sample than was available in our previous report of this finding (Wang & Voss, 2015).

In addition to indicating long‐lasting memory improvements due to stimulation, the between‐group difference for subjects receiving active versus sham stimulation during the first week of the experiment also supports the validity of the sham condition. That is, subjects were unlikely to have had knowledge of their stimulation condition during the first week of the experiment as comparison to the other condition would only have been possible during the second week. Thus, memory enhancement selective for the subjects receiving stimulation during the first week suggests that this effect was due to stimulation rather than due to knowledge of stimulation versus sham conditions. Notably, null effects on episodic memory have also been identified using a variety of control stimulation conditions of other brain areas (Kim et al., 2018; Nilakantan et al., 2017; Wang et al., 2014).

Mechanisms for long‐lasting effects of stimulation remain unknown. Although the same stimulation regimen used here has increased new episodic learning for ~24 hr after the final stimulation session in several other experiments using different test formats (Kim et al., 2018; Nilakantan et al., 2017, 2019), the long‐lasting (>1 week) effects of stimulation were less robust in those experiments than in the current replication experiment and in Wang et al. (2014). It is therefore possible that the current face‐cued word recall task is particularly sensitive to the effects of stimulation on plasticity of the hippocampal‐cortical network. Another possibility is that stimulation effects on episodic memory might also cause subjects to adopt specific memorization strategies that are beneficial for the current task, and that these memorization strategies persist over the between‐week delay period. For instance, changes in hippocampal–cortical network function due to stimulation could increase spontaneous adoption of effective memorization strategies that depend on this network, such as unitization or imagination of relationships between paired associates (Caplan & Madan, 2016; Rey et al., 2018; Staresina & Davachi, 2010). Subjects may then continue to use these effective strategies even after long between‐week delay periods. Future research could address this possibility by assessing memorization strategies or by using methods such as incidental encoding to limit the use of such strategies. Although mechanisms for long‐lasting stimulation effects are unknown, the current findings support the utility of the within‐subjects counterbalanced experiment design with week‐specific baseline assessments. That is, despite long‐lasting effects, subjects in this replication experiment and in Wang et al. (2014) only exhibited memory improvement after stimulation was delivered (Figure 2), and computing week‐specific change scores for the entire sample (Figure 1) accurately conveys the effects of stimulation on memory.

In summary, the current findings replicate those from a previous experiment (Wang et al., 2014) by identifying persistent episodic memory benefits of stimulation targeting the hippocampal–cortical network. Furthermore, the current findings do not support the proposal that increased episodic learning due to stimulation comes at the price of accelerated long‐term forgetting. Considered with other conceptual replications (Hermiller et al., 2019; Kim et al., 2018; Nilakantan et al., 2017, 2019; Tambini et al., 2018), the current results support the notion that noninvasive stimulation targeting the hippocampal–cortical network can influence recollective memory tasks that are widely considered hippocampal‐dependent.

## CONFLICT OF INTEREST

None declared.

## Data Availability

The data that support the findings of this study are available from the corresponding author upon reasonable request.

## Appendix 1

**Table A1:** List of verbal paired associates used for each version of the long‐term forgetting task. Subjects were required to recall the second item in each pair, shown in bold font, when prompted with the first item

Task Version 1
ROPE—**SMALL**
LAKE—**TRACK**
RENT—**TRICK**
HUMOR—**TAIL**
FREE—**CLOCK**
JUICE—**PLANE**
BREAK—**IMAGE**
RICH—**TREE**
GLORY—**MESS**
BATH—**FRUIT**
ROLL—**TRADE**
SOUND—**FORM**
PRICE—**FAVOR**
CLAIM—**WHEEL**
COLD—**HALL**
MILK—**BANK**
BUILD—**PHOTO**
PROUD—**FAULT**
RAIN—**CABLE**
CIVIL—**FACT**
BOSS—**OCEAN**
TRUTH—**WIND**
FAIRY—**CARDS**
LINE—**SPORT**
WOOD—**EXIST**
HANDS—**SHAPE**
PAINT—**SOLVE**
PILL—**SUIT**
Task Version 2
FIELD—**MEAL**
BRAIN—**BAND**
MOVIE—**ROCK**
BOAT—**PIPE**
FARM—**MOON**
FEET—**JUDGE**
EGGS—**PRIZE**
GOLF—**SMILE**
CAMP—**LEGAL**
ZONE—**QUIET**
PROOF—**STUCK**
BASIC—**SILLY**
HOOK—**STUDY**
PAPER—**BUNCH**
SWORD—**VOTE**
ANGER—**FUEL**
BOXES—**COLOR**
BRIDE—**SNOW**
PEACE—**LEADS**
WARN—**CLIMB**
TRAP—**FLAT**
HURT—**FISH**
CLASS—**POOL**
EYES—**WORD**
RADIO—**FEVER**
CROWD—**SALAD**
SERVE—**SPELL**
DOUGH—**NOISE**
